# Diagnostic value of glypican-1; a new marker differentiating pulmonary squamous cell carcinoma from adenocarcinoma: immunohistochemical study on Egyptian series

**DOI:** 10.1007/s10238-024-01551-5

**Published:** 2025-01-11

**Authors:** Iman Adel, Heba A Mahmoud, Amira Ismail Khater, Fatma S Hafez

**Affiliations:** 1 Oncologic Pathology Department, National Cancer Institute, Cairo University, Cairo, Egypt; 2 Pathology Department, Faculty of Medicine, Ain Shams University, Cairo, Egypt; 3 Cancer Epidemiology and Biostatistics Department, National Cancer Institute, Cairo University, Cairo, Egypt

**Keywords:** Glypican-1, Immunohistochemistry, Lung adenocarcinoma, Squamous cell carcinoma

## Abstract

Lung cancer is one of the major causes of cancer morbidity and mortality. Subtyping of non-small cell lung cancer is necessary owing to different treatment options. This study is to evaluate the value of immunohistochemical expression of glypican-1 in the diagnosis of lung squamous cell carcinoma (SCC). This retrospective study included a total of 68 cases, of which 36 were diagnosed as SCC and 32 as adenocarcinoma (ADC). Furthermore, glypican-1 expression was compared with the expressions of p63, thyroid transcription factor-1 (TTF-1), and napsin A. All cases of SCC except one showed positive immunostaining to glypican-1; 35/36 (97.2%) cases, and predominantly scored 3 + . While only 5 cases of ADC showed positive immunostaining to glypican-1, having a score of 1 + or 2 + . The difference between glypican-1 expression of the two tumor types was highly significant (*p* value < 0.001). The sensitivity, specificity, and overall accuracy of glypican-1 expression for differentiating lung SCC from ADC were 97.2%, 84.4%, and 91.2%, respectively. The sensitivity of glypican-1 is more than p63 in the diagnosis of lung SCC. Glypican-1 can be added as a new diagnostic marker to help in the accurate discrimination between poorly differentiated lung SCC and solid predominant adenocarcinoma cases.

## Introduction

Lung cancer is one of the major causes of cancer morbidity and mortality, accounting for about 12.4% of malignancies diagnosed worldwide and about 18.7% of cancer deaths. This disease ranks first among men and second among women, behind breast cancer, in terms of both incidence and mortality [1]. In Egypt, lung cancer is one of the most lethal malignancies. It is one of the top ten malignancies in both sexes, as it is the fifth most common cancer in men and relatively less common in women [2].

Lung cancer is a highly heterogeneous disease with a wide variety of clinicopathological and molecular characteristics [3]. Non-small-cell lung cancer (NSCLC) contributes to around 85% of all lung cancer cases. NSCLC can be further subdivided into two most prevalent subtypes, lung adenocarcinoma (ADC) and lung squamous cell carcinoma (SCC), which accounts for 50–60% and 20–30% of total NSCLC cases, respectively [3, 4].

Regarding SCC, smoking habits are the most well-known risk factor [5]. The incidence of SCC has been decreasing globally since 1979, despite an apparent increase recently reported, most likely due to the widespread use of immunohistochemistry for the classification of lung cancer histopathologic types and the subsequent decrease in not otherwise specified NSCLC diagnosis [6].

Over the last decade, immunotherapy and targeted therapy, among other treatments, have shown promising results in the treatment of advanced lung cancer, particularly NSCLC. They increased patients’ overall survival rates. This is mostly owing to the availability of biomarkers for selecting patients for targeted and immunotherapy-based treatments. As a result, such advances necessitate accurate subtyping of NSCLC [7–9].

Following the current therapeutic strategy algorithm for lung cancer patients, the diagnosis, including subtyping of lung cancer, is now linked directly to treatment of choice, but this subtyping is sometimes challenging especially when there are small biopsies and when the tumor is poorly differentiated [10, 11].

Glypicans, of the heparan sulfate proteoglycans (HSPGs) family, are attached to the plasma membrane outer surface via a glycosyl-phosphatidylinositol (GPI) anchor and act as growth factors [12]. Human glypican-1 (GPC1) is expressed in the central nervous system and skeletal system during development and also in other tissues of the adult [13].

Glypican-1 is overexpressed and has a diagnostic and prognostic value in multiple cancer types, including breast cancer, esophageal SCC (ESCC), glioma, and pancreatic cancer [14–18]. Its high expression is associated with poorer prognosis, making it a possible target for cancer therapy [17, 19]. In addition to its role in carcinogenesis, GPC1 can act as a coreceptor for multiple signaling molecules known for regulation of cell growth, motility, and differentiation [17].

Glypican-1 facilitates binding of fibroblast growth factor (FGF) to its receptor as it functions as a FGF coreceptor, and so fostering FGF-FGFR activation and signaling. As a result, receptor dimerization and transphosphorylation of tyrosine kinase domains occur, activating various signaling pathways, such as Ras-MAPK, PI3K-AKT-mTOR, and DAG-PKC. All signaling cascades ultimately promote cellular growth and survival as well as tumor angiogenesis [20, 21].

Additionally, GPC1 acts as a coreceptor of vascular endothelial growth factor-A (VEGF-A) which is an angiogenic growth factor crucial for angiogenesis. Exogenous GPC1 was shown to enhance VEGF-A/VEGFR binding in cell binding assays through employing endothelial cells, indicating a possible function for GPC1 in the regulation of angiogenesis [22, 23].

The transforming growth factor-*β* (TGF-*β*) is a cytokine that controls angiogenesis, inflammation, immunological response, differentiation, cell growth, and death by activating protein kinase receptors on the plasma membrane. Variable diseases, including cancer, are influenced by the dysregulation of this pathway. TGF-*β* and its receptors have been demonstrated to interact with GPC1 to stabilize their assembly for facilitated Smad signaling [24].

Moreover, GPC-1 functions as coreceptor to potentiate Wnt signaling pathway, which is important for many physiological and pathological processes, including embryonic development, differentiation, cell polarity, and tumor formation [25].

Several studies have investigated the possibility of using GPC1 as a therapeutic target in the treatment of solid tumors. Since inhibiting GPC1 activity using an anti-GPC1 monoclonal antibody has shown potential anti-tumor effects in a GPC1-positive ESCC xenograft model, it is expected that GPC1 targeted therapies can be developed for other GPC1-positive solid tumors [18].

This retrospective study aims at evaluating the immunohistochemical expression of GPC1 in diagnosis of lung SCC and its utility to differentiate between lung SCC and ADC.

## Material and Methods

### Tissue Samples

The histopathologic reports of cases of the study were randomly retrieved from the information network system of the Pathology Department of the National Cancer Institute (NCI)-Cairo University, Egypt, from January 2021 to December 2023. They fulfilled the inclusion criteria of the study, which are, in brief, cases with a primary diagnosis of NSCLC, only SCC and ADC subtypes, with available paraffin blocks that contained adequate, non-necrotic, and representative tumor tissue, and with available clinicopathological data in the computer database of the Pathology Department, while other histopathological types of NSCLC and small cell lung cancer as well as metastatic tumors to the lung were excluded. Clinicoradiologic correlation is necessary for exclusion of metastasis in addition to the available immunohistochemistry results. A total of 68 cases, divided as 36 SCC and 32 ADC, were included in this study. The relevant clinicopathological data as well as immunohistochemical (IHC) markers used to help in reaching the diagnosis were collected from the reports.

The conventional hematoxylin and eosin (H&E)-stained slides of all tumor cases were reviewed to verify the histopathological tumor type and grade, to confirm the presence of adequate tumor tissue in the slide fit to do and interpret the IHC staining, and to choose the best formalin-fixed paraffin-embedded (FFPE) tumor block in cases having multiple tumor blocks.

Criteria described in World Health Organization (WHO) classification of thoracic tumors, 5th edition [26] were applied to revise and reclassify lung tumors according to the histopathological findings.

### Glypican-1 Immunohistochemical Staining

The corresponding FFPE tumor tissue blocks of the included cases were retrieved from the archive of the Pathology Department of NCI, and one 4-*μ*m-thick unstained tissue section was cut with a microtome from the best representative FFPE tumor block of all cases, mounted on positively charged slides, and kept overnight in the oven in preparation for immunostaining.

The autoimmunostainer machine from Ventana vendor model type BenchMark ULTRA (Roche Diagnostics, Basel, Switzerland) was operated for IHC staining of GPC1 primary polyclonal antibody (catalog No. E-AB-40472, Elabscience, USA). Positive and negative control slides were processed with each patch of slides. A tissue section of a normal human kidney was used as a positive tissue control, while the negative control slide was processed by omitting the application of the antibody and adding only buffer.

### Assessment of Glypican-1 Immunostaining

The previously reported method by Kai et al. (2021) of GPC1 immunostaining interpretation was adopted in this study, where only cytoplasmic with or without cell membranous staining was considered positive and overlooking nuclear staining if any. The immunostaining positivity was scored as 1 + , 2 + , or 3 + if up to 10%, 10–50%, or > 50% of tumor cells showed positive immunostaining respectively [27]. The occasional presence of bronchial or bronchiolar epithelium helped as an internal positive control as it consistently stained positive for GPC1.

### Sample Size Calculation and Ethical Committee Approval

A total sample of 60 NSCLC cases was calculated with 5% absolute precision and 95% confidence. Sample size estimation was performed using Statulator (online statistical calculator).

The ethical approval to carry out the research was obtained from the members of the National Cancer Institute Review Board before the beginning of the study with the Institutional Review Board number (IRB No. PA2311-306–063).

### Statistical Considerations

Data management and analysis were performed using Statistical Package for Social Sciences (SPSS) version 27. Numerical data were summarized using mean and standard deviation or median (range), as appropriate. Categorical data were summarized as numbers and percentages. Student’s *t*-test was used to compare between two groups regarding normally distributed variables, while Mann–Whitney test was used to compare between two groups regarding non-normally distributed ones. Chi square or Fisher’s tests were used to compare between the groups with respect to categorical data, as appropriate. Sensitivity, specificity, positive predictive value (PPV), negative predictive value (NPV), and overall accuracy were calculated for GPC1 in all cases as well as for p63 in 44 cases. All tests were two-sided. *P*-values ≤ 0.05 were considered significant.

## Results

### Clinicopathological Characteristics

Our study included a total of sixty-eight cases, the mean age of all patients included in the study was 61.3 ± 8.4 years, ranging from 40 to 77 years, while the age of SCC patients ranges from 47 to 77 years, compared to 40 to 74 years in ADC patients. Interestingly, patients diagnosed with lung ADC were significantly younger than those having SCC, with patients’ mean age 58.2 ± 8.2 versus 64.0 ± 7.7, respectively, and this difference was statistically significant (*p* = 0.004). Detailed clinicopathological data, GPC1 expression, and other available IHC markers are shown in (Table 1).

**Table 1:** Clinicopathological characteristics of all studied lung SCC and ADC cases & overall IHC results of GPC1 and other used diagnostic markers (*n* = 68)

Characteristics		Number	Percent
Age (years)^a^		61.3 ± 8.4	
Sex	Male	58	85.3
	Female	10	14.7
Type of surgery	Biopsy	59	86.8
	Excision	3	4.4
	Lobectomy	6	8.8
Tumor grade	Grade 2	8	11.8
	Grade 3	60	88.2
Necrosis	Present	24	35.3
	Absent	44	64.7
Site of biopsy (*n* = 65)^b^	Bilateral	2	3.1
	Left	27	41.5
	Right	36	55.4
Bilaterality (at initial radiologic investigations)	Unilateral	63	92.6
	Bilateral	5	7.4
Histopathological type	Squamous cell carcinoma	36	52.9
	Adenocarcinoma	32	47.1
Glypican-1	Positive	40	58.8
	Negative	28	41.2
Score of glypican-1	0	28	41.2
	1 +	10	14.7
	2 +	11	16.2
	3 +	19	27.9
p63 (*n* = 44)	Positive	23	52.3
	Negative	21	47.7
TTF1^c^ (*n* = 48)	Positive	27	56.3
	Negative	21	43.8
Napsin A (*n* = 17)	Positive	10	58.8
	Negative	7	41.2

Data about p63, TTF1 and napsin A IHC staining were only available in 44, 48 and 17 reports, respectively

^a^ Variable is presented as mean ± standard deviation

^b^ Site of biopsy was not mentioned in three cases

^c^TTF1: Thyroid transcription factor-1

The cases were divided as regards histopathological types into thirty-six cases diagnosed as SCC and thirty-two cases as ADC. All cases of SCC were poorly differentiated, or grade 3, while ADC cases were predominantly grade 3 in 24 out of the 32 studied cases, and the remaining eight cases were of grade 2 (Table 2).

**Table 2:** Clinicopathological characteristics and GPC1 immunoreactivity scores in relation to tumor histopathological types (*n* = 68)

Characteristics*		Squamous cell carcinoma	Adenocarcinoma	*P* value
		*n* = 36	*n* = 32	
		*n* (%)	*n* (%)	
Age (years)^a^		64.0 ± 7.7	58.2 ± 8.2	**0.004**
Sex	Male	30 (51.7)	28 (48.3)	0.739
	Female	6 (60.0)	4 (40.0)	
Type of surgery	Biopsy	30 (50.8)	29 (49.2)	0.395
	Excision	3 (100.0)	0 (0.0)	
	Lobectomy	3 (50.0)	3 (50.0)	
Tumor grade	Grade 2	0 (0.0)	8 (100.0)	**0.001**
	Grade 3	36 (60.0)	24 (40.0)	
Necrosis	Present	12 (50.0)	12 (50.0)	0.720
	Absent	24 (54.5)	20 (45.5)	
Score of glypican-1	0	1 (3.6)^b^	27 (96.4)^c^	**< 0.001**
	1 +	7 (70.0)^b^	3 (30.0)^b^	
	2 +	9 (81.8)^b^	2 (18.2)^c^	
	3 +	19 (100.0)^b^	0 (0.0)^c^	

Bold indicates statistical significance

*Data are presented as number and row percentage

^a^Variable is presented as mean ± standard deviation

^b,c^Groups having different letters are significantly different from each other

### Glypican-1 IHC expression

**Fig. 1:**
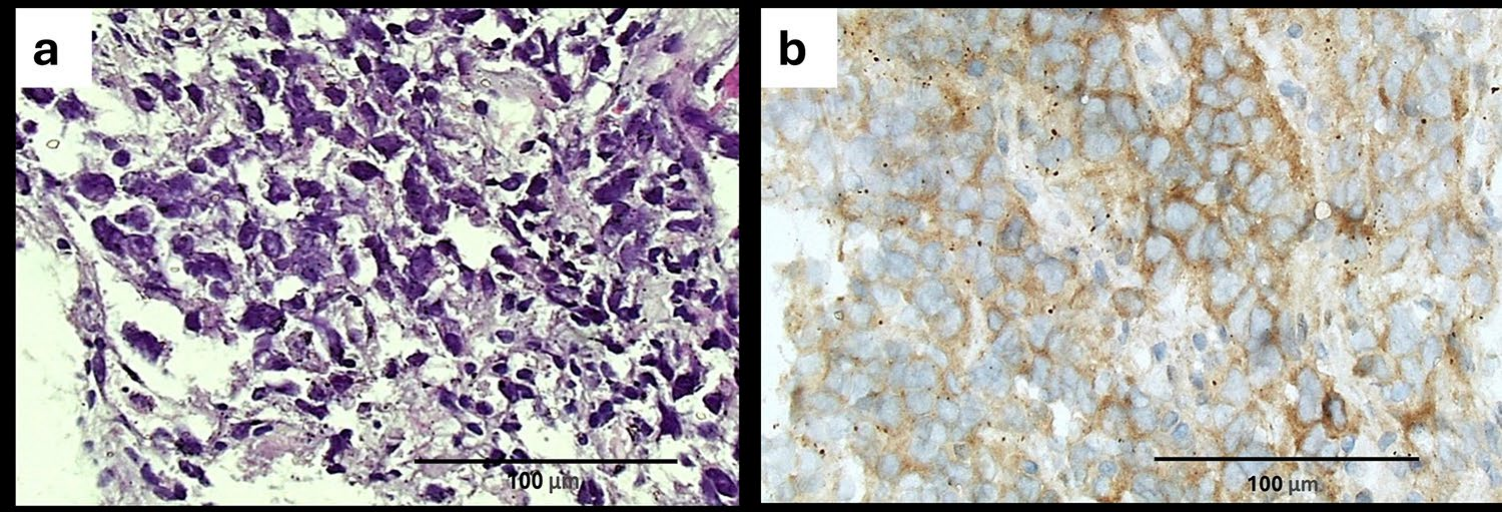
A case of lung poorly differentiated SCC exhibited score 3 + for GPC1. **a** The H&E-stained section showing the necessity of IHC marker to help in establishing the diagnosis (H&E, original magnification × 400). **b** Near all tumor cells showed positive cytoplasmic immunostaining to GPC1 (DAB, original magnification × 400)

**Fig. 2:**
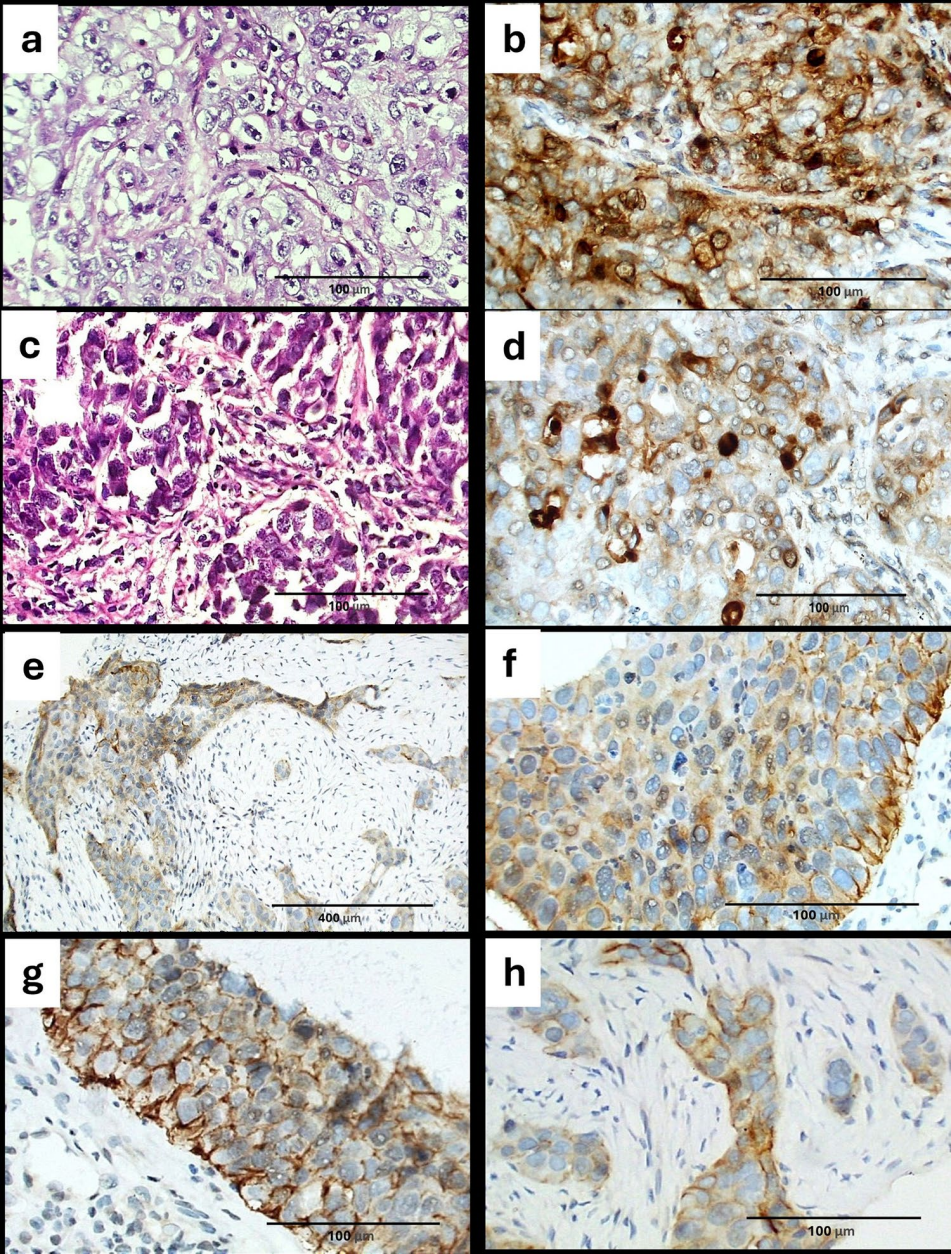
Representative sections from several cases of lung SCC stained by H&E and with GPC1 polyclonal antibody. **a** Case of non-keratinizing lung SCC grade 3 formed of solid sheets of tumor cells (H&E, original magnification × 400). **b** The same case immunoreacted strongly to GPC1 within cytoplasm of tumor cells and scored 3 + (DAB, original magnification × 400). **c** Another case of lung SCC exhibiting bizarre anaplastic nuclei and gland-like formations needs further verification by marker for definite evaluation (H&E, original magnification × 400). **d** The previous case showed strong cytoplasmic score 3 + GPC1 immunostaining reaction with cell membranous accentuation (DAB, original magnification × 400). **e** Another case of lung SCC showing heterogeneous GPC1 score 2 + immunostaining (DAB, original magnification × 100). **f**, **g**, **h** Higher magnification of previous case (DAB, original magnification × 400)

**Fig. 3:**
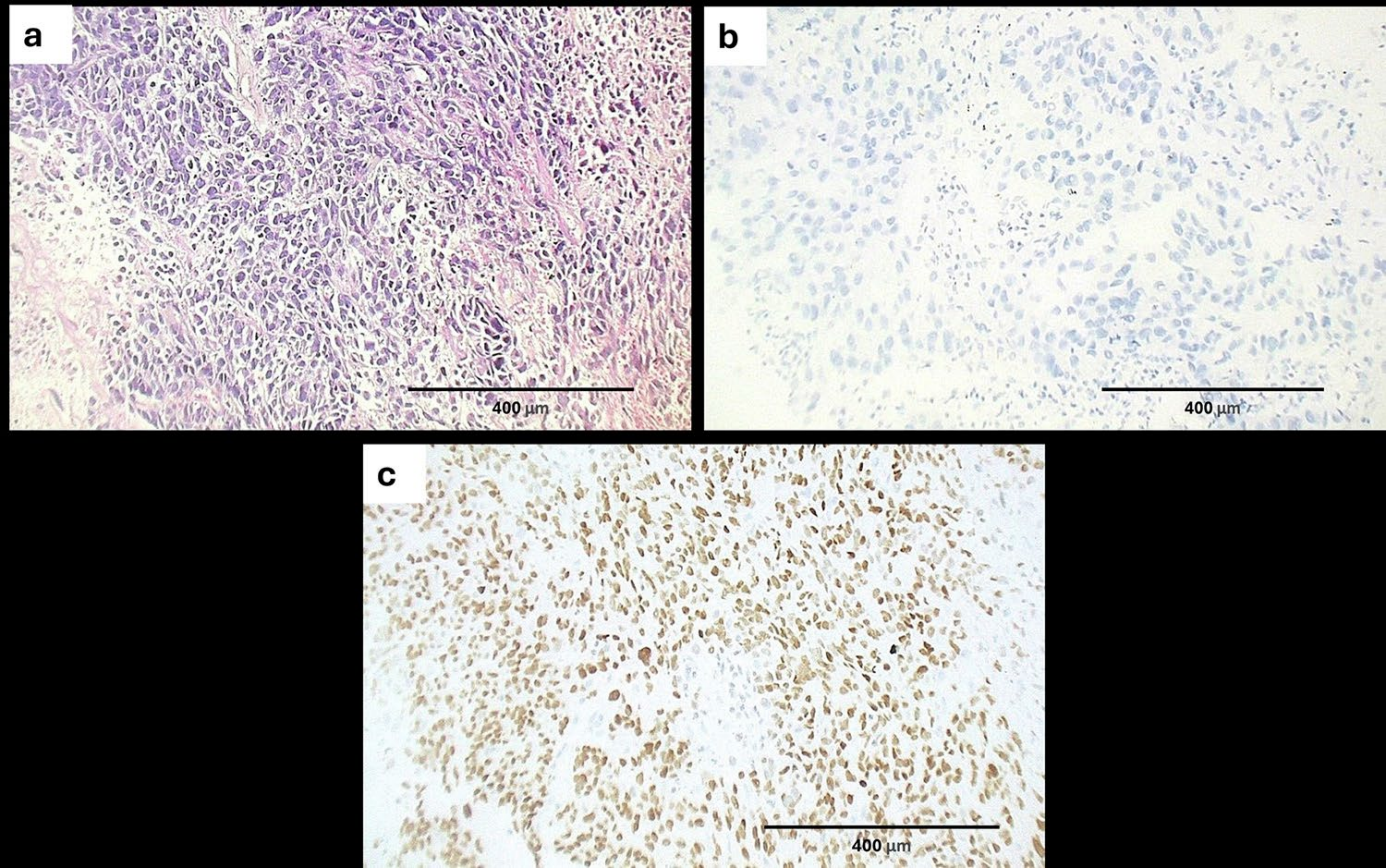
A case of lung poorly differentiated ADC **a** Sheets of tumor cells with areas of necrosis (H&E, original magnification X 100), **b** The case showed negative GPC1 immunoreaction (DAB, original magnification × 100), **c** The tumor cells showed positive TTF1 immunostaining confirming the diagnosis of ADC of lung origin (DAB, original magnification × 100)

All cases of SCC except one showed positive immunostaining reaction to GPC1; 35/36 (97.2%), compared to only 5 out of 32 (15.6%) ADC cases showing positive GPC1 immunostaining. None of these 5 GPC1-positive ADC scored 3 + but rather scored either 1 + in three cases or 2 + in the remaining two cases. Contrary, the 35 GPC1-positive SCC predominantly scored 3 + , followed by score 2 + and least scored 1 + ; 19, nine and seven cases, respectively. Obviously, higher GPC1 scores were more related to SCC, while score (0) was more related to ADC. GPC1 immunoexpression has been significantly related to the histopathological type of NSCLC, where positive expression of GPC1 as well as its positive immunostaining scoring was related to SCC histopathology, while negative GPC1 immunostaining was related to ADC. This relationship yielded a highly significant *p* value (< 0.001) (Table 2) (Figs. 1, 2, and 3).

Data about p63 immunostaining was available in 44 histopathology reports, where cases were IHC stained with p63 antibody, within which 23 cases were p63 positive, where all except one were of SCC type and one was ADC. Twenty-one cases were p63 negative, including two SCC and 19 ADC. Thyroid transcription factor-1 (TTF1) IHC staining was reported in 48 out of the 68 studied cases, and it was positive in 27 cases, all of which were of ADC type, and negative in 21 cases, all of which were of SCC type. Napsin A immunostaining was reported in only 17 cases, and it was positive in 10/17 cases; all were ADC and negative in seven cases; all were SCC (Tables 1 and 3).

**Table 3:** Immunohistochemical findings for lung squamous cell carcinoma and adenocarcinoma (*n* = 68)

Tumor marker*		Squamous cell carcinoma	Adenocarcinoma
Glypican-1 (*n* = 68)	Positive	35	5
	Negative	1	27
p63 (*n* = 44)	Positive	22	1
	Negative	2	19
Thyroid transcription factor-1 (TTF1, *n* = 48)	Positive	0	27
	Negative	21	0
Napsin A (*n* = 17)	Positive	0	10
	Negative	7	0

*Data are presented as number of cases

Regarding the relationships of GPC1 immunostaining with clinicopathological characteristics of the studied cases, it was found that GPC1 immunostaining pattern in terms of positive or negative was significantly related to the age of patients, with the mean age of patients whose tumors were GPC1 positive being significantly older than those whose tumors were GPC1 negative (63.5 years, ranging from 47.0 to 77.0 years, versus 58.0 years, ranging from 40.0 to 74.0 years, respectively), with a significant relationship (*p* = 0.045). However, GPC1 immunostaining was not significantly related to the sex of patients (*p* = 0.507) (Table 4).

**Table 4:** Glypican-1 in relation to different clinicopathological characteristics (*n* = 68)

Characteristics*		Glypican-1		*P* value
		positive	Negative	
		*n* (%)	*n* (%)	
Age (years)^a^		63.5 (47.0–77.0)	58.0 (40.0–74.0)	**0.045**
Sex	Male	33 (56.9)	25 (43.1)	0.507
	Female	7 (70.0)	3 (30.0)	
Tumor grade	Grade 2	0 (0.0)	8 (100.0)	**< 0.001**
	Grade 3	40 (66.7)	20 (33.3)	
Necrosis	Present	15 (62.5)	9 (37.5)	0.649
	Absent	25 (56.8)	19 (43.2)	

Bold indicates statistical significance

*Data are presented as number and row percentage

^a^Variable is presented as median (range)

Meanwhile, GPC1 positive staining was not related to the presence or absence of tumor necrosis (*p* = 0.649). On the other hand, the relationship between GPC1 positive immunoreactivity and tumor grade was highly significant (*p* < 0.001), where all eight grade two cases included in the study were GPC1 negative (Table 4).

### Sensitivity, specificity, positive predictive value, negative predictive value and diagnostic accuracy of glypican-1 with insight on p63 marker

The sensitivity, specificity, PPV, and NPV were calculated for GPC1 IHC marker in the 68 studied cases of NSCLC, and it was found that the sensitivity of GPC1 was 97.2%, specificity was 84.4%, PPV was 87.5%, NPV was 96.4%, and overall accuracy was 91.2%. The same was calculated for p63, showing that GPC1 was more sensitive but less specific than p63 in the diagnosis of lung SCC: 97.2% versus 91.7 in sensitivity and 84.4% compared to 95% in specificity, respectively. While the PPV of GPC1 was less than that of p63, and its NPV surpassed that of p63. The overall diagnostic accuracy of GPC1 in the diagnosis of lung SCC and differentiating it from lung ADC was approaching that of p63 (91.2%) versus (93.2%), respectively (Table 5).

**Table 5:** Sensitivity, specificity, PPV, NPV and diagnostic accuracy rate of glypican-1 and p63 IHC markers for the differential diagnosis of lung SCC from lung ADC (n = 68)

Tumor marker	Sensitivity (%)	Specificity (%)	PPV (%)	NPV (%)	Overall accuracy (%)
Glypican-1	97.2	84.4	87.5	96.4	91.2
p63 (n = 44)^a^	91.7	95.0	95.7	90.5	93.2

PPV, positive predictive value; NPV, negative predictive value

^a^Data about p63 IHC marker was available in 44 reports out of the 68 cases of the study

## Discussion

Discrimination between lung SCC and ADC is very crucial for the planning of gene-targeted therapy strategies. In this regard, studies aimed to assess the expression of novel markers that have a role as a newly developed gene-targeted therapy in different tumors, to verify the possibility of using them on the studied tumors [21].

Normal human tissues show limited GPC1 expression, so it can be used as a suitable target for antibody-based targeted therapy. A reported preclinical result supports the concept of using GPC1 as a cancer antigen for potential therapeutic regimens [28]. Moreover, studies have emerged to verify the possibility of using GPC1-targeted therapy in the treatment of other solid malignancies expressing it [21].

In the current study, GPC1 positivity was observed in 97.2% of lung SCC cases (35 out of 36). The IHC expression score in most of it (54.3%) was 3 + , while expression scores of 2 + and 1 + were found in 25.7% and 20.0% of the positive cases, respectively. That was in contrast to ADC cases, where GPC1 IHC expression was detected in 15.6% of cases (5 out of 32), with expression scores of 1 + and 2 + in three cases (60.0%) and two cases (40.0%), respectively. Regarding the IHC expression of GPC1 in the two tumor types, it was significantly higher in lung SCC compared to ADC.

To the best of our knowledge, few studies have been conducted to evaluate the diagnostic utility of GPC1 IHC expression in the differentiation between lung SCC and ADC. Our results are concordant with the study conducted by Kai et al. (2021) that revealed strong positive expression of GPC1 in 100% of poorly differentiated lung SCC cases with scores of 3 + and 2 + in 96.8% and 3.2% of their cases, respectively, compared to score 1 + in only 3.3% of poorly differentiated ADC cases [27]. The same results were obtained by El-Naby et al. (2023) study, which found positivity for GPC1 in 100% of lung SCC cases with high expression in 80% of them and positive expression in only 6.7% of lung ADC cases, which was focal and weak [29]. So, we can conclude that a score of 3 + for GPC1 expression is the most valuable for the diagnosis of SCC, followed by scores of 2 + and 1 + .

The IHC profile obtained for ADC in this study is comparable with what was obtained by Amatya et al. (2018). They found GPC1 positivity in only 3% of ADC cases, which was weak and focal (score 1 +) [30]. Abdelrahman et al. (2024) also found a low and weak positive expression of GPC1 in ADC of the lung (6.7%) [31].

In the current study, different portions of cases have been assessed by other IHC markers that were used in the panel of differentiation between SCC and ADC. Assessment of p63 expression revealed positivity in 92% of the SCC cases compared to only 5% of ADC cases. TTF1 and napsin A were positive in all ADC cases and negative in all cases of SCC.

These results are in concordance with Amatya et al. (2018) study, which confirmed the results by assessing the expression of TTF1 and napsin A in ADC cases, and it reported a positivity of 89% and 84%, respectively [30].

As regards the relation between GPC1 expression and patients’ characteristics, this study revealed a significant relationship between GPC1 expression and patients’ age. This could be attributed to the significant difference between the mean age of SCC cases and ADC cases being higher in SCC, in which GPC1 shows positivity.

Moreover, the relation between positive GPC1 IHC staining and tumor grade was highly significant. But this shouldn’t be interpreted as a real difference, as we have selected cases focusing on poorly differentiated tumors that need further IHC markers to establish a definite diagnosis.

No significant relation was detected between GPC1 expression and other clinicopathological criteria. A previous study revealed a significant relation between GPC1 expression and some histopathological data like tumor size, grade, stage, and lymph node metastasis [29]. Another study reported a significant relation between GPC1 expression and tumor size and lymph node metastasis [32]. This disagreement between our results and theirs could be attributed to a difference in sample sizes.

Referring to the differentiation between lung SCC and ADC in the current study, GPC1 sensitivity and specificity were high, 97.2% and 84.4%, respectively. This is similar to a study that reported a GPC1 sensitivity of 100%, specificity of 96.7%, and diagnostic accuracy of 98.4% [27].

In relation to p63 in this study, both GPC1 and p63 have comparable overall accuracy, with slightly higher GPC1 sensitivity. P63 is a well-established marker in the confirmation of SCC diagnosis [33]. Kai et al. (2021) also reported high GPC1 sensitivity and specificity for the same purpose, which were in very close values compared to p40 expression and higher values compared to CK 5/6 expression [27]. P40 represents the positive marker with a good accuracy in SCC diagnosis, and CK 5/6 is another positive marker used for the same differentiation issue [27].

The overall diagnostic accuracy of GPC1 as a new marker was approaching that of p63, with the advantage of higher sensitivity and NPV over that of p63. This may suggest that both markers can be used to help in the differential diagnosis of the difficult, poorly differentiated cases of NSCLC, helping to distinguish SCC. This adds the GPC1 marker to the list of markers that can help in revealing the exact tumor cell lineage of NSCLC, especially in poorly differentiated cases not identified by the conventional stain and need further IHC studies.

## Conclusion

GPC1 has proved its overall diagnostic accuracy in the diagnosis of lung SCC that was comparable to the other well-established p63 IHC marker used in the differential diagnosis of NSCLC differentiating lung SCC from lung ADC. Therefore, it can be added as a new diagnostic marker to help in the accurate discrimination of difficult, poorly differentiated lung SCC from solid predominant ADC cases that need IHC investigation. In this regard, GPC1 can help as a positive marker for lung SCC and a negative marker for lung ADC. Moreover, GPC1 could have a promising role as a targeted therapy for lung SCC in the future.

## Data Availability

No datasets were generated or analyzed during the current study.
